# Rheumatoid nodule presenting as an indeterminate soft tissue mass in the sole of the foot

**DOI:** 10.1093/jscr/rjad278

**Published:** 2023-05-27

**Authors:** Ravi Patel, Raghav Nand, Dakshinamurthy Sunderamoorthy

**Affiliations:** Department of Trauma and Orthopaedics, The Princess Royal Hospital, Telford, UK; Department of Trauma and Orthopaedics, Robert Jones and Agnes Hunt Orthopaedic Hospital, Oswestry, UK; Department of Trauma and Orthopaedics, Scunthorpe General Hospital, Scunthorpe, UK; Department of Trauma and Orthopaedics, Scunthorpe General Hospital, Scunthorpe, UK

## Abstract

A 64-year-old lady with a background of rheumatoid arthritis presented to the foot and ankle clinic with lump underneath the sole of her foot causing significant discomfort. Examination revealed she had a swelling of the first and the second metatarsophalangeal joints. Magnetic resonance imaging revealed abnormal soft tissue thickening between the second and the third metatarsal and a single large encapsulating indeterminate soft tissue mass with a peripheral inflammatory rim. The appearance was suggestive of a malignant sarcoma rather than a rheumatoid nodule or rheumatoid tenosynovitis. The patient was referred to the regional sarcoma unit where the scans were reviewed, and a sarcoma was ruled out. The patient then underwent excision of the indeterminate soft tissue mass. Histology revealed granulomatous infiltration suggestive of a rheumatoid nodule. This has not been described previously in the literature.

## INTRODUCTION

Rheumatoid nodules are well-demarcated, flesh coloured, subcutaneous lumps occurring in a quarter of patients with rheumatoid arthritis [1]. The most common sites include areas with pressure and occur adjacent to joints on extensor surfaces, such as the elbow, fingers and forearms [2]. Plantar involvement is rare and seldom described in the literature; nodules are generally observed in the context of multiple lesions in other locations [3]. This case report aims to inform doctors that a rheumatoid nodule should be considered when dealing with unknown soft tissue masses in patients with rheumatoid arthritis. More importantly, unique cases such as this should be discussed in a regional sarcoma unit before excision is considered.

## CASE REPORT

A 64-year-old lady with a background of rheumatoid arthritis presented to the foot and ankle clinic with lump underneath the sole of her foot causing significant discomfort. She had a background of anticitrullinated protein antibody positive rheumatoid arthritis treated by oral methotrexate (MTX). In the past she had a surgical fusion of the right foot first metatarsophalangeal joints (MTPJ) as a Weil’s osteotomy of the second toe metatarsal. On examination she had painful soft tissue mass swelling over the first and the second MTPJ cystic in consistency.

Magnetic resonance imaging revealed abnormal soft tissue thickening between the second and third metatarsal and a single large encapsulating indeterminate soft tissue mass with a peripheral inflammatory rim. The appearance was suggestive of a malignant sarcoma rather than a rheumatoid nodule or rheumatoid tenosynovitis. Laboratory test showed systemic inflammation with C-reactive protein levels of 64 mg/l (reference range <5 mg/l), normal serum urate (170 umol/L, reference range 160–355umol/L) and blood cultures were negative.

**Figure 1:**
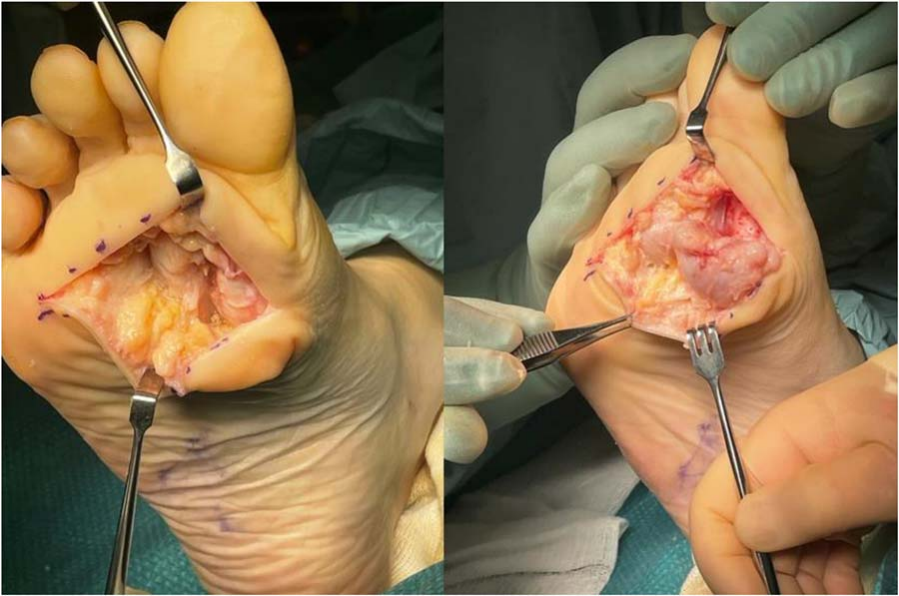
Large, poorly circumscribed nodule, difficult to separate from surrounding soft tissues.

**Figure 2:**
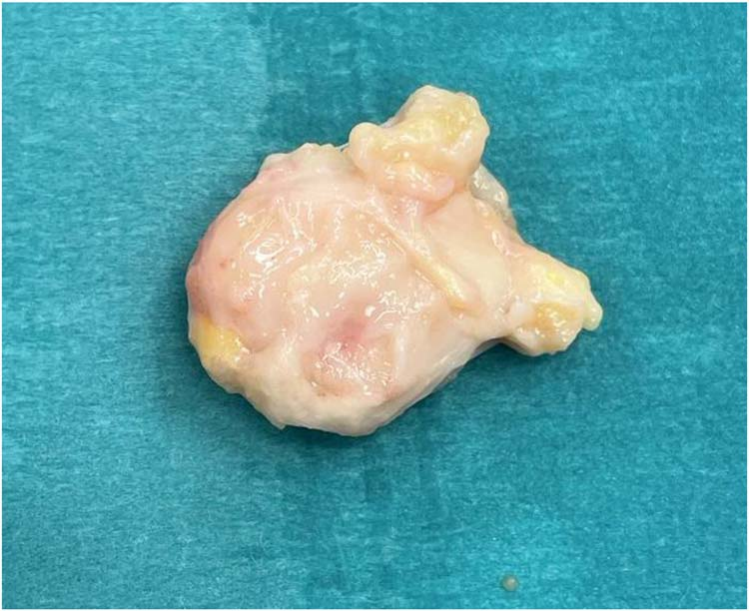
Excised mass was excised with soft tissue pedicles, measuring ~4.0 cm × 3.0 cm.

The patient was referred to the regional sarcoma unit where the scans were reviewed, and a sarcoma was ruled out. The patient underwent surgical excision of the indeterminate soft tissue mass. Intraoperatively, a large soft tissue mass 4.0 × 3.0 cm in diameter involving the digital nerve and capsule of the second MTPJ was noted, along with necrosis of the underlying fat pad (Figs 1and 2).

Histopathology concluded the specimen consisted granulomatous infiltration with peripheral palisading and no evidence of features specific to a neuroma, dysplasia or malignancy. The presence of granulomatous lesions was indicative of a rheumatoid nodule with local infection.

Post-operatively at the 3 month follow-up the patient was able to ambulate pain free with no distal neurovascular compromise. She was subsequently discharged from the clinic.

## DISCUSSION

Rheumatoid nodules occur in ~20% of all adult rheumatoid arthritis patients [1–3]. Most patients with rheumatoid nodule are rheumatoid factor-positive, and are known to have a worse prognosis, less frequent symptom relief and more frequent extra-articular symptom manifestations [4]. Similar subcutaneous nodules are also observed in other diseases, such as gout, amyloidosis, rheumatoid fever, sarcoidosis, xanthoma and erythematous nodules [4–6]. Rheumatoid nodules, mainly in women, are known to occur between skin and bony prominences that are frequently in friction, such as the olecranon of the ulna, the proximal part of the ulna, the lateral side of the fingers, the gluteal or Achilles tendon and the femoral process. It occurs frequently on the ischial seat, etc. [4]. Although the mechanism of development is not well known, it is speculated to be the result of an autoimmune reaction by T lymphocytes [6]. Although the natural course of rheumatoid nodules is generally benign and grows slowly, some cases of rapid size increase are known after systemic immunosuppressant administration [5, 6]. The most common clinical symptom is a palpable soft tissue mass, which may be accompanied by pain. Diseases that need to be clinically differentiated include ganglion, xanthoma, gout and soft tissue abscess, and when continuous systemic immunosuppressive drugs are used for the treatment of rheumatoid arthritis, lymphoma or soft tissue sarcoma should also be included in the differential diagnosis [7]. Pathologically, rheumatoid nodules can be divided into three distinct structural segments. Fibrinous necrosis is located in the centre, inflammatory cells showing a desk arrangement surround the outside and the outermost part is composed of granulation tissue [1, 3, 4].

On plain radiograph, the subcutaneous rheumatoid nodule appears as a lobular expansive soft tissue mass. Lesions rarely contain calcifications, and it can be a differentiating point from a mass. In addition, it is known that cases of bone erosion caused by rheumatoid nodule are rare [1]. Recently, in cases of rheumatoid arthritis, magnetic resonance imaging, which has been widely used to identify abnormalities in joints and synovial membranes, can also be used to evaluate abnormalities in soft tissues around the joints. The findings of magnetic resonance imaging of both nodules were presented.

El-Noueam *et al*. [6] classified rheumatoid nodules in five patients into two categories based on magnetic resonance imaging findings, and the entire mass showed high signal intensity when contrast enhancement was performed, and second, a case with peripheral contrast enhancement showing high signal intensity on the fat suppression T2-weighted image and including the central portion without contrast enhancement. Divided pathologically, in the former, the entire mass was composed of chronic inflammatory cells and small blood vessels, while in the latter the centre was composed of necrosis, and there was infiltration of inflammatory cells and fibrotic cells around it.

Starok *et al*. [5] reported magnetic resonance imaging findings of four rheumatoid nodules on the hand of a patient. On T1-weighted images, these nodules had low to moderate signal intensities and appeared heterogeneous. On T2-weighted images, the two relatively small nodules showed signal intensities similar to those of the surrounding muscles, but the medium-sized nodule showed lamellar inhomogeneous signal intensity, and the largest nodule had very inhomogeneous signal intensity, with internal borders containing parts of high signal intensity that were easily erased. These nodules showed different enhancement patterns: the medium-sized nodule showed the strongest enhancement, the small nodule showed weak enhancement and the largest nodule had no enhancement in the internal cystic part and only the periphery was enhanced. This case showed similar findings to the largest nodule. The diversity of magnetic resonance imaging findings of rheumatoid nodules is probably due to suppuration of the nodule [8].

Magnetic resonance imaging findings of rheumatoid nodules are not specific, but considering the patient’s history and clinical features, the width can be narrowed, and it can be used to evaluate the range before surgery during surgery. Indications for rheumatoid nodule surgery include pain, progressive neurological abnormalities, decreased joint mobility, cosmetic problems and differentiation from soft tissue masses.

In this case, there was a history of rheumatoid arthritis, but the symptoms were not severe, and it was clear that the rheumatoid factor was positive, and an abnormal soft tissue thickening between the second and the third metatarsal and a single large encapsulating indeterminate soft tissue mass with a peripheral inflammatory rim was detected on magnetic resonance imaging.

It was a mass with suggestive findings and other nonspecific findings, making it difficult to differentiate it from synovial sarcoma or soft tissue sarcoma such as malignant fibrous histiocytoma. The possibility of granulomatous synovitis caused by tuberculosis may be considered a differential and proliferative pigmented villous nodular synovitis. In addition, even if the symptoms are not severe, it is considered that rheumatoid nodule should have been included in the differential diagnosis considering the medical history. Isdale *et al*. [9] presented a case of rheumatoid nodule, which was difficult to differentiate from soft tissue sarcoma in the left lower leg. It was an invasive mass that diffusely invaded the anterior compartment of the lower leg on CT.

## CONCLUSION

In patients reporting pain and presenting with an indeterminate soft tissue mass located in the sole of the foot, we urge clinicians to consider the referral to the regional sarcoma unit to rule out possibility of sarcoma as the primary diagnosis.

Although not specific, rheumatoid nodules can be used for the differential diagnosis of solid or well-circumscribed subcutaneous tissue masses with internal necrosis that occur in areas where there is a lot of friction between bone and skin in patients with rheumatoid arthritis.

This case is rare, at the time of writing there are only a handful of published papers detailing a rheumatoid nodule presenting as an indeterminate soft tissue. Our hope is that this information can be used by surgeons to inform their decisions when referring to the regional sarcoma unit and improve patient outcomes.
